# The promiscuous biotin ligase TurboID reveals the proxisome of the T3SS chaperone IpgC in *Shigella flexneri*

**DOI:** 10.1128/msphere.00553-24

**Published:** 2024-10-31

**Authors:** Nathaline Haidar-Ahmad, Kyle Tomaro, Mathieu Lavallée-Adam, François-Xavier Campbell-Valois

**Affiliations:** 1 Department of Chemistry and Biomolecular Sciences, Centre for Chemical and Synthetic Biology, Host-Microbe Interactions Laboratory, University of Ottawa, Ottawa, Ontario, Canada; 2 Department of Biochemistry, Microbiology and Immunology, Centre for Infection, Immunity and Inflammation, University of Ottawa, Ottawa, Ontario, Canada; 3 Department of Biochemistry, Microbiology and Immunology, Ottawa Institute of Systems Biology, University of Ottawa, Ottawa, Ontario, Canada; The University of Iowa, Iowa City, Iowa, USA

**Keywords:** Enterobacteria, *Shigella flexneri*, type III secretion system, IpgC, MxiE, transcription regulation, protein-protein interactions, cell signaling, promiscuous biotin ligase, BirA, TurboID, BioID

## Abstract

Promiscuous biotin ligases derived from the bacterial enzyme BirA are used to identify proteins vicinal to a bait protein, thereby defining its proxisome. Despite the popularity of this approach, surprisingly little is known about its use in prokaryotes. Here, we compared the activity of four widely used promiscuous biotin ligases in the cytoplasm of *Shigella flexneri*, a pathogenic subgroup of *Escherichia coli*. Our data indicate that the kinetics of TurboID’s biotinylating activity is the highest of those tested. In addition, TurboID showed reduced interaction with the natural BirA binding partners, BccP and the biotin operator, when compared to its ancestor BioID. We therefore evaluated the ability of TurboID to probe the proxisome of the type III secretion system (T3SS) chaperone IpgC and the transcriptional activator MxiE. When the T3SS is inactive (off-state), these proteins are inhibited by forming complexes with the T3SS substrates OspD1 and IpaBC, respectively. In contrast, when the T3SS is active (on-state), OspD1 and IpaBC are secreted allowing MxiE and IpgC to interact together and activate their target genes. The results obtained with the IpgC and TurboID fusions capture a good fraction of these known interactions. It also suggests that the availability of IpgC increases in the on-state, resulting in a greater number of proteins detected in its vicinity. Among these is the T3SS ATPase SpaL (also known as Spa47 or SctN), further supporting the notion that chaperones escort their substrate to the T3SS. Interestingly, a specific subset of proteins conserved in *E. coli* completes the IpgC proxisome in the on-state.

**IMPORTANCE:**

Promiscuous biotin ligases are widely used to study protein function in eukaryotes. Strikingly, their use in prokaryotes has been rare. Indeed, the small volume and the cytoplasmic location of the biotin ligase’s natural binding partners in these organisms pose unique challenges that can interfere with the study of the proxisome of proteins of interest. Here, we evaluated four of the most common promiscuous biotin ligases and found TurboID was best suited for use in the cytoplasm of *Shigella flexneri*. Using this method, we extended the proxisome of IpgC beyond its known direct binding partners involved in the regulation of the type III secretion system (T3SS) signaling cascade. Of particular interest for further study are transcription factors and housekeeping proteins that are enriched around IpgC when the T3SS is active. We propose a model in which the increased availability of IpgC in the on-state may allow cross-talk of the T3SS with other cellular processes.

## INTRODUCTION

*Shigella* spp. are members of the family Enterobacteriaceae, the second leading cause of death from diarrheal diseases with 200,000 cases in 2016 (1), and on the World Health Organization’s priority list for the development of new vaccines and antibiotics (2, 3). They are divided into four subgroups named *boydii*, *dysenteriae*, *sonnei*, and *flexneri,* the latter being the most common cause of disease, especially in developing countries (4, 5). *Shigella* strains are distributed in several phylogroups of *Escherichia coli* (6), and are characterized by a large invasion plasmid encoding a type III secretion system (T3SS) (7).

The T3SS gives *Shigella* the ability to invade the cytoplasm of epithelial cells, which is the primary site of bacterial replication (8–11). The T3SS translocates protein substrates from the bacterial cytoplasm to the host cell cytoplasm. Among its substrates, the translocators IpaB and IpaC and the tip complex protein IpaD play a critical role in the formation and the rupture of the *Shigella*-containing vacuole, which are necessary for *Shigella* cytoplasmic invasion (12). A critical feature of the T3SS is the regulation of the secretion of its substrates. Indeed, the T3SS is turned on upon contact with the host plasma membrane and remains active during the maturation of the *Shigella*-containing vacuole (13). When the vacuole is finally ruptured, the T3SS is switched off and remains so, as long as the bacterium is in the cytoplasm. This switching of the T3SS between its off- and on-states is repeated each time a new cell is infected (14). Importantly, the knockout of *ipaB* or *ipaD* can be used to mimic the on-state in microbiological medium in the absence of host cells (15, 16).

The *Shigella* T3SS is also the receptor of a rather unique signaling pathway. Upon physical contact with its agonist, the plasma membrane, the tip complex is thought to undergo a conformational change that is transmitted along the needle to the export apparatus to activate secretion (17–19). This results in as much as a 30- to 50-fold increase in the transcription of genes encoding approximately 20 T3SS substrates (20, 21), termed late substrates B (7, 12), which include the IpaH E3 ubiquitin ligase family (22). Their expression requires a MxiE binding box in their promoter, the transcriptional activator MxiE and its co-activator IpgC (20, 23, 24), and is described by what will be referred to here as the T3SS signaling model. It proposes that the tight repression of these genes in the off-state is based on the titration of MxiE and IpgC in inhibitory complexes formed with OspD1 and its chaperone Spa15 and IpaB or IpaC, respectively. However, these inhibitory complexes are disrupted when the T3SS is active because OpsD1 and IpaBC are secreted, thereby allowing the now freed cytoplasmic IpgC and MxiE to form a complex that activates gene expression (25). The formation of these protein complexes is not equally well understood. IpgC is the chaperone of IpaB and IpaC, and their interaction is supported by biochemical and structural evidence (26, 27). The formation of the MxiE-OspD1-Spa15 and MxiE-IpgC is supported by epistasis and coelution from affinity columns (23, 25, 28), but their structures are unknown. Furthermore, to our knowledge, the involvement of IpgC and MxiE in other cellular processes has not been reported.

Variants of the bacterial biotin ligase BirA with promiscuous biotinylation activities, such as BioID, BioID2, miniTurbo, and TurboID, are used to study protein complexes *in situ* (29–32). They release the reactive intermediate biotin-AMP, which spontaneously reacts with primary amines within a radius of 10 to 35 nm of the active site (32–34). When these promiscuous biotin ligases are fused to a bait protein, the vicinal proteins are labeled and their identification is used to define the bait proximity proteome or “proxisome.” This information can then be further exploited to determine whether vicinal proteins are functionally connected to the bait. Promiscuous biotin ligases are widely used in mammalian tissue culture cells or animal models (35, 36), including some studies that have used them to identify protein complexes formed by bacterial effectors inside host cells (37, 38). However, proxisome studies using promiscuous biotin ligases have been rare in bacteria (39, 40). A sensitive issue is the native function of BirA, which is to synthesize biotin-5′-AMP and use it to biotinylate the biotin carboxylase BccP. Under biotin-rich conditions, when BccP is fully biotinylated, BirA associated with biotin-5′-AMP forms a homodimer that binds to the biotin operator to repress biotin synthesis genes (41). Interference from these native functions could reduce the performance of BirA-derived promiscuous biotin ligases in proxisome studies. This problem is particularly acute when studying cytoplasmic proteins, as BccP and DNA are located in the cytoplasm. In particular, the lack of compartments and the small volume of the bacterial cytoplasm may exacerbate the problem. In our study, we found that TurboID is suitable for application in the cytoplasm of *Shigella* and used it to identify proteins in the vicinity of MxiE and IpgC when the T3SS is in the off- or on-state. The proxisome of IpgC confirmed several interactions with proteins involved in the T3SS signaling cascade, as well as putative novel cross-talks with proteins that are not commonly associated with the T3SS. This study provides valuable insights for future applications of promiscuous biotin ligases in prokaryotes.

## RESULTS

### TurboID catalyzes rapid promiscuous biotinylation in the cytoplasm of *S. flexneri*

**Fig 1:**
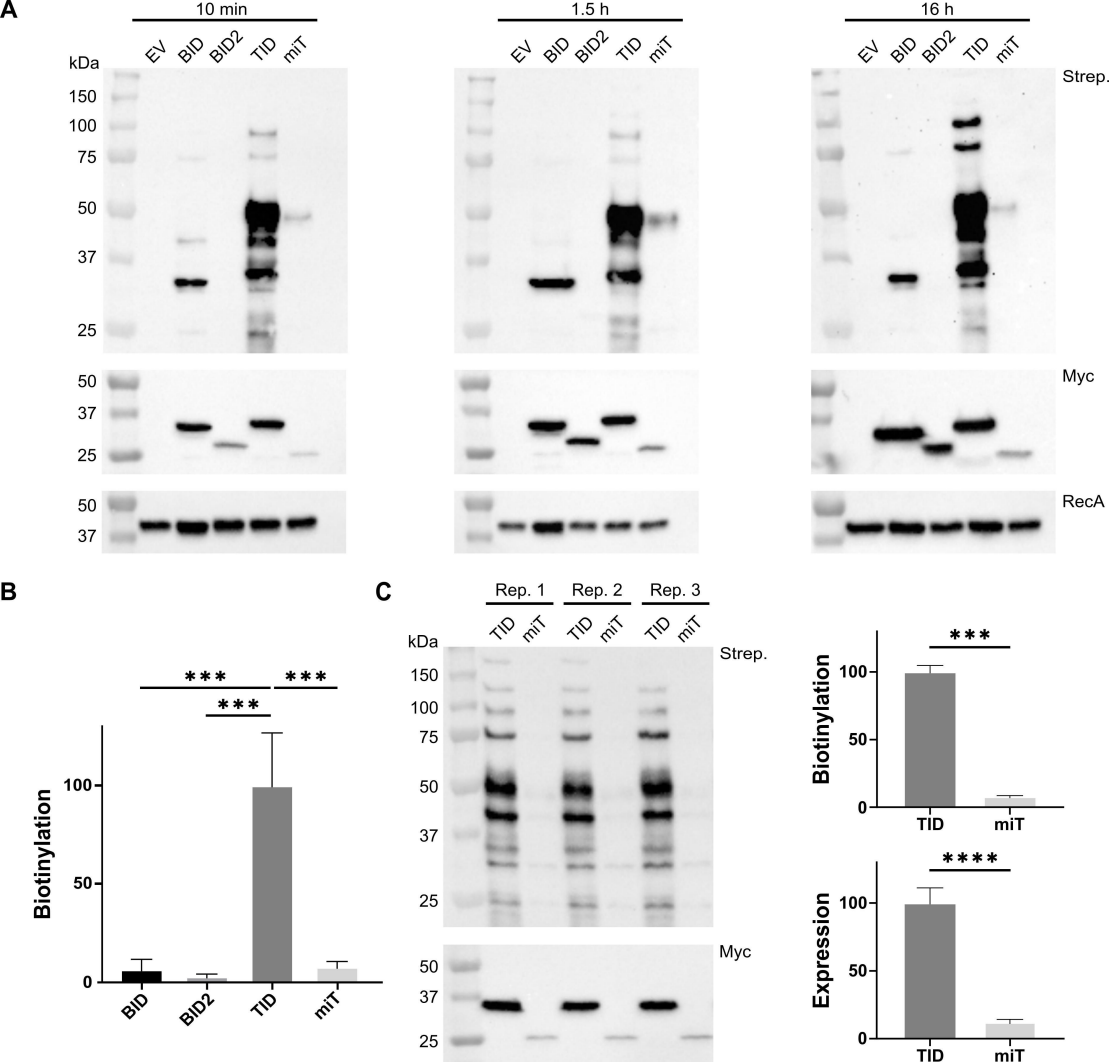
TurboID is a highly effective promiscuous biotin ligase in the cytoplasm of *Shigella flexneri*. (**A**) Biotinylation was measured at the indicated times after the addition of 50 µM biotin to cultures of *S. flexneri* strain M90T harboring the empty vector (EV), BioID-Myc (BID), BioID2-Myc (BID2), TurboID-Myc (TID), miniTurbo-Myc (miT). pSU2.1 was used as the backbone vector. The expression of the biotin ligases and the loading controls was measured by immunoblotting using a monoclonal antibody against the Myc tag and a polyclonal antibody against RecA, respectively. The biotinylation blots were performed with the streptavidin-horseradish peroxidase (Strep.). (**B**) Densitometry on three biological replicates (*n* = 3) of the biotinylation blot represented in panel A was used to estimate the relative biotinylation activity of the biotin ligases at 10 min after the addition of biotin. The results of a one-way analysis of variance with Tukey’s *post hoc* test are shown (***, *P* < 0.001). Error bars represent standard deviation to the mean. (**C**) Biotinylation and Myc blots for a new set of three biological replicates (*n* = 3) labeled Rep. 1, 2, or 3, comparing TurboID to miniTurbo 10 min after the addition of biotin. The graphs show the densitometry data for the biotinylation activity (Strep., top panel) and expression (Myc, bottom panel), respectively. The results of Student’s *t*-tests for unpaired data with equal variance and a 95% CI are shown (***, *P* < 0.001; ****, *P* < 0.0001). Error bars represent standard deviation to the mean.

First, we compared the bystander biotinylation activity of the free promiscuous biotin ligases BioID, BioID2, TurboID, and miniTurbo when expressed in the cytoplasm of *Shigella flexneri* strain M90T using a streptavidin-horseradish peroxidase biotinylation blot and measured their production by immunoblotting with a Myc tag antibody (Fig. 1A). We monitored the activity of the biotin ligases at 10 min, 1.5 hours, and 16 hours after the supplementation of the medium with 50 µM biotin. We observed similar relative activities of the biotin ligases at all these time points, with TurboID consistently coming out on top. This was followed by BioID and miniTurbo, whereas BioID2 was barely detectable under these conditions. Nevertheless, when tested separately, we readily detected BioID2’s biotinylation at 16 hours (data not shown), suggesting that it is active in the cytoplasm of *S. flexneri*. We then used densitometry to compare the activity of the biotin ligases at 10 min using three independent biological replicates (Fig. 1B). Only TurboID yielded highly reproducible labeling that was approximately 20-fold superior to the runner-up BioID. In particular, the lower production of BioID2 and miniTurbo is a major determinant of their lower activity. These differences in expression levels were consistent with observations in eukaryotes (31, 32). The removal of the N-terminal domain in miniTurbo, which is conserved in TurboID and BioID, is probably responsible for its lower production. Nevertheless, miniTurbo has previously been used in the bacterial cytoplasm. Therefore, we sought to accurately compare its activity with that of TurboID. To do this, we obtained three new biological replicates that we probed on the same membrane to reduce the likelihood of technical error (Fig. 1C). Using densitometry, we estimated the activity of TurboID 10 min after adding biotin to be 13.2 ± 2.8 times greater than that of miniTurbo. After correcting for the expression levels measured with the Myc tag, TurboID was 1.6 ± 0.5 more active than miniTurbo, which is consistent with the previous estimate in eukaryotes at 30°C (32). Therefore, the low production of miniTurbo is the dominant factor underlying its poor performance in *S. flexneri* at 37°C. We decided to pursue BioID and TurboID as they seemed the most promising for immediate application in this organism.

### TurboID has partly lost the native functions of its ancestor BirA

**Fig 2:**
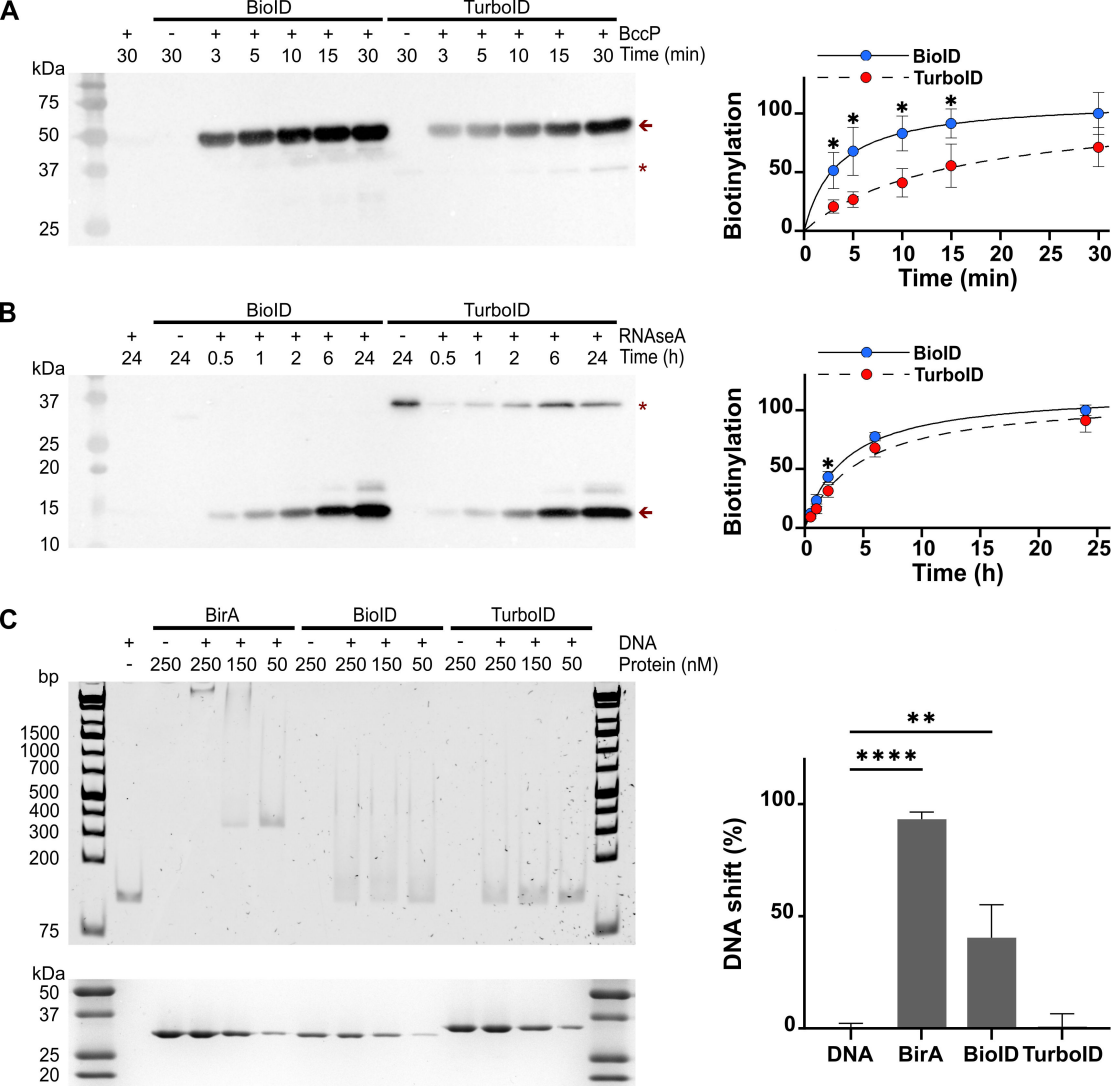
TurboID has partially lost the native functions of its ancestor BirA. Biotinylation of MBP-BccP by 6×His-BioID and 6×His-TurboID in function of time (**A**). The arrow indicates MBP-BccP. The star represents autobiotinylation of the biotin ligases and the unlabeled bands are contaminants. Densitometry of three biological replicates (*n* = 3) of the BccP biotinylation blot were used to generate this timecourse graph (righ panel). The results of Student’s *t*-tests for unpaired data with equal variance and a 95% CI are shown (*, *P* < 0.05). Error bars represent standard deviation to the mean. The datapoints were fitted with a rectangular hyperbola, indicating that the midpoint of biotinylation (*t*^50^) was 3.4 min and 14.6 min for BioID (*R* = 0.99) and TurboID (*R* = 0.99), respectively. Biotinylation of RNase A by 6×His-BioID and 6×His-TurboID in function of time (**B**). The arrow indicates RNase A. The star represents autobiotinylation of the biotin ligases and the unlabeled bands are contaminants. Densitometry of three biological replicates (*n* = 3) of the RNase A biotinylation blot were used to generate this timecourse graph (right panel). The results of Student’s *t*-tests for unpaired data with equal variance and a 95% CI are shown (*, *P* < 0.05). Error bars represent standard deviation to the mean. The datapoints were fitted with a rectangular hyperbola, indicating that *t^50^* was 3.4 hours and 4.7 hours for BioID (*R* = 0.99) and TurboID (*R* = 0.99), respectively. (**C**) Electrophoretic mobility shift assay showing the DNA binding properties of BirA, BioID, and TurboID. To perform this assay, 40 nM of a 112 bp DNA probe containing the biotin operator was mixed with 250, 150, and 50 nM of biotin ligases in the presence of excess biotin (1 µM) and ATP (1 mM). The protein input is shown in the Coomassie-stained SDS-PAGE gel in the bottom panel. Three biological replicates of this experiment (*n* = 3) were used to estimate the shift of the DNA probe in the presence of 50 nM of each of the indicated biotin ligases (right panel). The results of a one-way analysis of variance with Dunnett’s *post hoc* test are shown (**, *P* < 0.01; ****, *P* < 0.0001). Error bars represent standard deviation to the mean.

BioID and TurboID are both derived from the *E. coli* strain K-12 BirA biotin ligase. Since *Shigella* BirA and BccP are identical to their *E. coli* K-12 orthologs, they may be able to titrate BioID and TurboID cytoplasmic bait fusions away from the intended targets. Therefore, a crucial question is whether BioID and TurboID can be effectively used for proxisome studies in *Shigella* despite retaining some native BirA functions. First, we compared the ability of BioID and TurboID to biotinylate BccP *in vitro*. This activity requires forming a stable complex with biotin-5′-AMP, whereas promiscuous biotinylation relies on the release of biotin-5′-AMP. We hypothesized that the more promiscuous TurboID would have a reduced ability to biotinylate BccP compared to BioID. To test this, we purified BioID, TurboID, and BccP, the natural substrate of BirA, and conducted an *in vitro* biotinylation reaction. The data suggest that the activity of TurboID toward BccP is indeed reduced, as indicated by its slower rate of biotinylation compared to that of BioID (Fig. 2A). In contrast, the activity of both enzymes against the RNase A was similar (Fig. 2B). The other main function of BirA is to bind the biotin operator under a homodimeric form when biotin is in excess. Due to their promiscuous nature, we reasoned that the complex of the promiscuous enzymes with biotin-5′-AMP should be less stable than that formed by their ancestor BirA, thereby preventing the formation of the biotin ligase homodimer that binds the biotin operator. In addition, TurboID-specific mutations may also disrupt the homodimer interface. To test this hypothesis, we performed an electrophoretic mobility shift assay between BirA, BioID, and TurboID and the biotin operator DNA probe in the presence of excess biotin and ATP (Fig. 2C). BirA at a concentration of 50 nM completely shifted the DNA probe, whereas the shift induced by BioID and TurboID in decreasing order was partial even at 250 nM. At 50 nM, BirA, BioID, and TurboID shifted 93%, 41%, and 0.8% of the DNA probe, respectively. These data confirmed that BioID and especially TurboID bound the biotin operator with lower affinity than BirA. Taken together, these data indicated that TurboID was the best candidate for studying protein complexes in the cytoplasm of *S. flexneri* and related *Enterobacteriaceae*.

### IpgC and MxiE-TurboID fusions are functional

We then constructed N-terminal and C-terminal TurboID protein fusions of IpgC and MxiE and measured their expression levels. We also evaluated their function using a complementation assay that measured their ability to rescue the expression of IpaH proteins in knockout strains lacking either *ipgC* or *mxiE* in strains with an otherwise wild-type (WT) genetic background in which secretion is inactive (T3SS in the off-state) and in strains Δ*ipaD* or *ipaB4* whose T3SS is active (T3SS in the on-state). Since the ability of MxiE and IpgC to activate the expression of the *ipaH* is enhanced in the on-state, one would expect the MxiE and IpgC-TurboID fusions, if they function normally, to rescue the expression of IpaHs.

**Fig 3:**
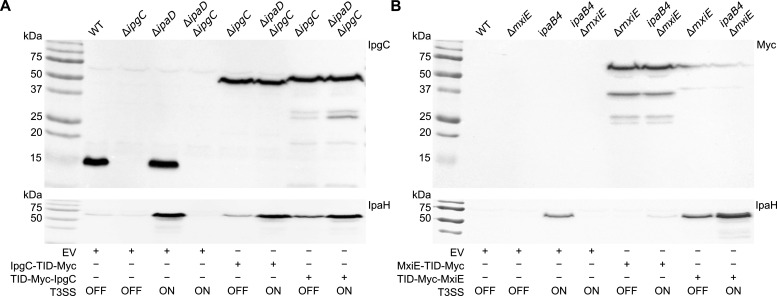
TurboID fusions with MxiE are partially functional, while those with IpgC are fully functional. Expression (top panel) and complementation assay (bottom panel) of IpgC and TurboID (TID) fusions compared to the empty vector in indicated *S. flexneri* strains with their T3SS in the off- or on-state (**A**). Expression (top panel) and complementation assay (bottom panel) of MxiE and TID fusions compared to the empty vector in indicated *S. flexneri* strains with their T3SS in the off- or on-state (**B**). pUC18.1rp was used as the backbone vector. The expression of TID fusions with IpgC and MxiE were measured by immunoblotting with a polyclonal antibody against IpgC and a monoclonal antibody against the Myc tag, respectively. The complementation assay consisted in measuring the rescue of IpaHs production by immunoblotting with a polyclonal antibody against IpaH9.8. These data are representative of three biological replicates (*n* = 3).

First, using a polyclonal antibody against IpgC, we found that a pUC18 derivative with the *rpsM* promoter allowed expression of the IpgC fusions in Δ*ipgC* and Δ*ipaD* Δ*ipgC* at levels comparable to the endogenous IpgC expressed by the WT and Δ*ipaD* (Fig. 3A, top panel). As expected, the expression of the IpaH proteins was low in the WT and higher in Δ*ipaD*, and was abrogated in Δ*ipgC* and Δ*ipaD* Δ*ipgC* (Fig. 3A, bottom panel). Interestingly, both IpgC-TurboID-Myc and TurboID-Myc-IpgC (IpgC-TurboID and TurboID-IpgC thereafter) rescued the expression of the IpaH. As expected, the IpaH production was higher in the Δ*ipaD* Δ*ipgC* (on-state) complemented strains than in the Δ*ipgC* (off-state) complemented strains. Notably, the expression of the IpaH in the latter was slightly higher than in the WT strain, indicating a moderate gain of function in the off-state. Since the expression of the protein fusions was similar to the endogenous IpgC, this phenomenon is probably due to a partial loss of the anti-co-activator activity of IpaBC against the IpgC fusions. Notably, TurboID-IpgC showed an additional product with a lower molecular weight than expected, suggesting partial degradation of this protein fusion. Despite these minor discrepancies, the overall data suggest that IpgC is functional when TurboID is fused to either of its termini.

In the absence of an MxiE antibody, we used a Myc tag to assess the production of the MxiE fusions (Fig. 3B, top panel). Full-length products were detected for both MxiE fusions, but degradative products were significant for the higher expressed MxiE-TurboID-Myc (MxiE-TurboID thereafter). Moreover, this fusion was slightly functional, as indicated by its poor rescue of the IpaH production in *ipaB4* Δ*mxiE* (Fig. 3B, bottom panel). By contrast, TurboID-Myc-MxiE (TurboID-MxiE thereafter) migrated as a single band and was able to rescue the production of IpaH in Δ*mxiE* (off-state) and *ipaB4* Δ*mxiE* (on-state) with the expected higher expression in the latter. However, IpaHs production was increased compared to the WT and *ipaB4*, suggesting that the amount of MxiE available to bind the *ipaH* promoters was increased in cells expressing TurboID-MxiE. This may be due to its overproduction or to a reduced ability of the anti-activator OspD1 to inhibit MxiE in this context. These data indicate that all protein fusions retain at least some of the functions of their endogenous counterparts with IpgC-TurboID and TurboID-MxiE providing the best functional rescue.

**Fig 4:**
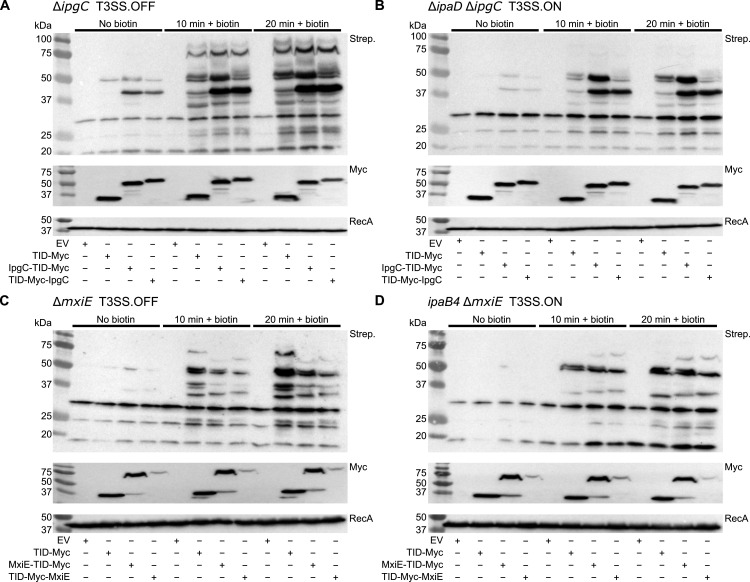
The biotinylation patterns of TurboID fusions with MxiE are indistinguishable from the free TurboID, whereas those with IpgC show a unique pattern. Biotinylation patterns of IpgC-TurboID (TID)-Myc and TID-Myc-IpgC compared to the empty vector (EV) and free TID-Myc in *S. flexneri* Δ*ipgC* (**A**), and Δ*ipaD* Δ*ipgC* (**B**) with the T3SS in the off-state and on-state, respectively. Biotinylation patterns of MxiE-TID-Myc and TID-Myc-MxiE compared to the EV and free TID-Myc in *S. flexneri* Δ*mxiE* (**C**), and *ipaB4* Δ*mxiE* (**D**) with the T3SS in the off-state and on-state, respectively. pUC18.1rp was used as the backbone vector. The expression of the biotin ligases and the loading control were measured by immunoblotting using a monoclonal antibody against the Myc tag and a polyclonal antibody against RecA, respectively. The biotinylation blots were performed with the streptavidin-horseradish peroxidase (Strep.). The effect of 50 µM biotin supplementation of the medium was probed at 10 min and 20 min and compared to samples in which no biotin was added. These data are representative of three biological replicates (*n* = 3).

We then compared the biotinylation patterns of TurboID fusions with IpgC and MxiE with that of the free TurboID (Fig. 4). First, we observed that the biotinylation induced by TurboID prior to the addition of biotin is significant. This is consistent with the performance of TurboID in yeast (32), suggesting that TurboID can utilize the basal biotin present in the medium to induce biotinylation. Following the addition of 50 µM biotin, protein biotinylation increased sharply at 10 min, with a further gradual increase at 20 min. By using densitometry to compare the band intensities at 10 min with biotin supplementation to the control without biotin supplementation, we estimated that 61% ± 17% and 68% ± 24% of the biotinylation happened after biotin supplementation in the Δ*ipgC* strain with IpgC-TurboID and TurboID-IpgC, respectively (Fig. 4A). Therefore, the majority of proteins are biotinylated in the last 10 min of the incubation. Second, the biotinylation pattern of IpgC-TurboID fusions differed from the free TurboID with unique bands located between 37 and 75 kDa in the strain with T3SS in the off-state (Fig. 4A). The proteins with molecular weight above 50 kDa decreased in strains with the T3SS in the on-state (Fig. 4B), suggesting that they may correspond to T3SS substrates. In contrast, the biotinylation patterns of TurboID fusions with MxiE were indistinguishable from that of the free TurboID (Fig. 4C and D). This is likely due to the masking of rare prey-specific biotinylation events by the background biotinylation of abundant proteins. Taken together, these data suggest that TurboID fusions are functional, i.e., they can form specific transcription-activating protein complexes directed by IpgC and, to a lesser extent, MxiE. Therefore, we set out to use these TurboID fusions to study the proxisome of IpgC and MxiE in the same strains described above to mimic the off- and on-states adopted by the T3SS during *Shigella* infection.

### The proxisome of IpgC reveals an increase in the diversity of vicinal proteins when the T3SS is active

Biotinylated proteins were purified on a streptavidin resin and identified by tandem mass spectrometry. We used spectral counts and the Significance Analysis of INTeractome (SAINT) software (42) to identify proteins that were significantly enriched in the TurboID fusions with IpgC and MxiE compared to the TurboID and empty vector controls (Tables S1 and S2). Nevertheless, the list of proteins filtered by the SAINT analyses still contained three major classes of likely false positives. First, proteins involved in translation, such as ribosomal proteins, are common contaminants in mass spectrometry due to their abundance or their proximity to baits during their biosynthesis. Second, the major naturally biotinylated protein BccP and proteins involved in biotin synthesis BioB and BioD are contaminants that are expected with promiscuous biotin ligases when used in the uncompartmentalized bacterial cytoplasm. Finally, T3SS substrates, especially those whose expression is regulated by MxiE and IpgC, are a class of false positives that is specific to this biological system. Indeed, the concentration of T3SS substrates in the cytoplasm may vary as a function of their secretion and expression. Under our experimental conditions, the expression of MxiE-regulated T3SS substrates is superior in *ipgC*-complemented strains expressing the TurboID fusion than in the control uncomplemented strain expressing the TurboID alone. In addition, IpgC and MxiE may also be vicinal to the protein product of their target genes as translation is co-transcriptional in bacteria. Due to the sensitivity of proximity labeling to the cellular concentration of individual proteins, all of these factors can lead to false-positive identification of T3SS substrates in the vicinity of IpgC or MxiE.

**Fig 5:**
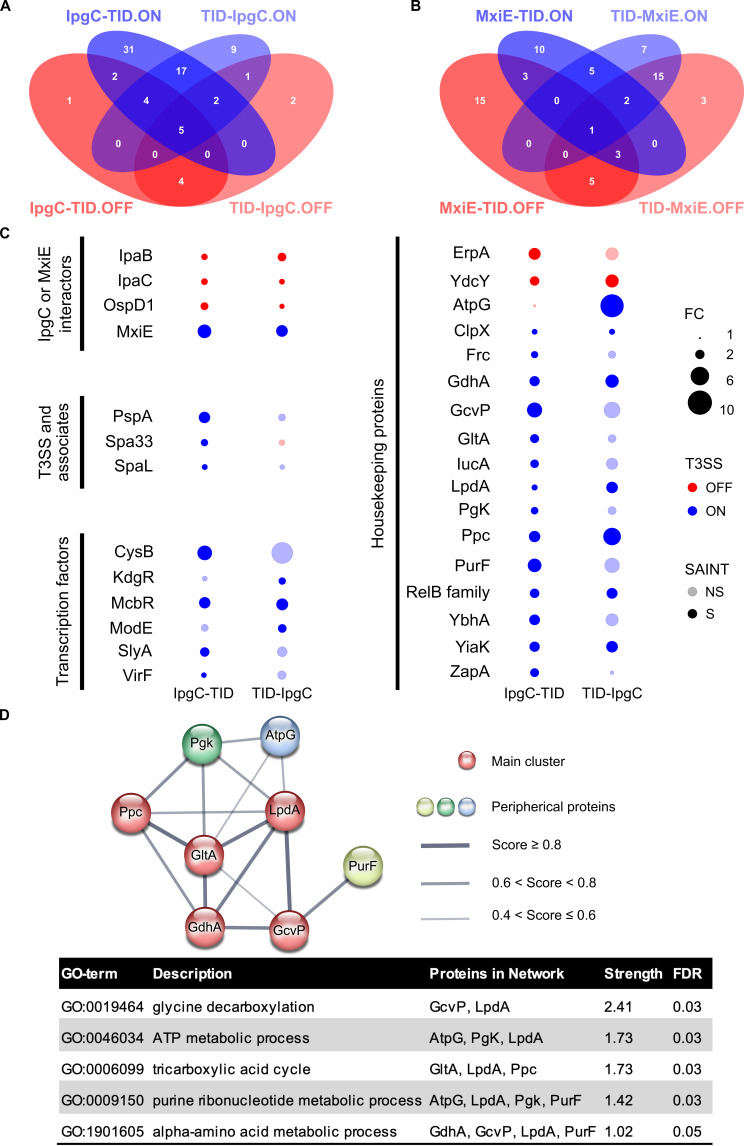
TurboID identifies the proxisome of IpgC and MxiE. Venn diagrams showing the overlap between the proteins identified in the off-state and on-state of the T3SS for both fusions of (**A**) IpgC (*n* = 5) and (**B**) MxiE (*n* = 6) with TurboID (TID). (**C**) Dot plot representation of the fold change (FC) and SAINT significance [false discovery rate (FDR) < 10%] for high-confidence protein hits identified in the IpgC-TurboID-Myc (IpgC-TID) or TID-Myc-IpgC (TID-IpgC) experiments. Fold change >1 is off/on and on/off for proteins that are more abundant in the off-state (blue) and on-state (red), respectively. The proteins are organized into four protein groups: IpgC or MxiE interactors, T3SS and associates, transcription factors, and housekeeping proteins. (**D**) The STRING network formed by a subset of housekeeping proteins identified in panel C. The width of the edges indicates the confidence in the predicted interactions, which can be direct (physical) or indirect (functional). The wider edges have the highest confidence. The table shows the five most enriched Gene Ontology (GO) terms (FDR ≤ 5%) in this network and their associated proteins. Note: LpdA is named lpd in the STRING database; YiaK is also known as DglD; RelB family is a protein with homology with the RelB and DinJ antitoxins.

For IpgC, the number of proteins enriched in the on-state was superior to those in the off-state (Fig. 5A). IpgC’s known binding partners IpaB, IpaC, and MxiE were enriched in both TurboID fusions, suggesting this approach identified some of IpgC’s direct binding partners, which are expected to constitute a substantial fraction of its proxisome (Table S3). For MxiE, the number of proteins identified in both states was the same (Fig. 5B). Among its known binding partners, OspD1 was identified, but not IpgC (Table S4). Since the IpgC data were consistent with the *Shigella* T3SS signaling model, we decided to pursue the analysis of its proxisome in greater details.

From the remaining candidates shortlisted by SAINT (Fig. 5A; Table S3), we selected a subset of high-confidence hits that included T3SS-associated proteins suspected to interact with IpgC based on the literature, and proteins not previously associated with IpgC that had a fold change (FC) (on/off or off/on) >2 with at least one of the TurboID fusions. These data are presented in a dot plot organized into four groups of proteins: IpgC or MxiE interactors, the T3SS and associates, transcription factors, and houskeeping proteins (Fig. 5C; see Table S3 for fold change numerical values). IpaB and IpaC were slightly enriched in the off-state (1 < FC_off/on_ < 2), whereas MxiE was enriched in the on-state (2.7 > FC_on/off_ < 3.5), as predicted by their direct interaction with IpgC in the T3SS signaling model. However, the enrichment of OspD1 in the off-state (1.2 > FC_off/on_< 1.6) was unexpected as OspD1 is supposed to bind MxiE, but not IpgC (25). It is noteworthy that the proximity of IpgC with these proteins was deemed of high confidence by SAINT with both protein fusions (false discovery rate [FDR] < 10%).

Most of the other proteins in the IpgC proxisome are enriched in the on-state. They include the ATPase SpaL (FC_on/off_ ≈ 1.3), which is a cytoplasmic component of the T3SS that forms the substrate sorting platform, and the phage shock protein A, PspA, (FC_on/off_ ≈ 2.7) whose cotranscribed PspB and PsbC are associated with T3SS secretin-stress tolerance (43). Notably, these proteins, which belong to the T3SS and associates’ group, are confidently identified by SAINT (FDR < 10%) only with the IpgC-TurboID fusion. Beyond MxiE, other transcription regulators were found in the vicinity of IpgC in the on-state. They included the transcription activator VirF (FC_on/off_ ≈ 1.2), which is the master regulator of the T3SS regulatory cascade (7). Furthermore, five chromosomal transcriptional factors were enriched in the on-state. McbR (FC_on/off_ ≈ 2.7), SlyA (FC_on/off_ ≈ 2.0), and CysB (FC_on/off_ ≈ 4.0) are the most confidently identified based on SAINT and their label-free quantification (LFQ) intensities (Fig. 5C; Table S3). Finally, the IpgC proxisome is completed by a group of housekeeping proteins, most of which are metabolic enzymes and are enriched in the on-state. According to the STRING database, some of these enzymes formed a highly connected network with an average node degree of four and a protein-protein interaction (PPI) enrichment *P*-value of 3.2 × 10^−9^ (44) (Fig. 5D). In addition, five gene ontology (GO) terms were enriched (FDR ≤ 5%). However, there is no evidence that any pair of these proteins directly binds to each other.

## DISCUSSION

In this study, we have determined the proxisome of the chaperone IpgC in the off- and on-state of the T3SS. The data confirmed known binding partners and revealed an array of additional proteins that are found in the vicinity of IpgC, particularly when the T3SS is in the on-state. This study is one of the first applications of promiscuous biotin ligases to study the proxisome of proteins in the bacterial cytoplasm. Therefore, it can be used to guide future works that will harness this powerful approach.

We expected that the use of promiscuous biotin ligases in the bacterial cytoplasm would be challenging. Indeed, the lack of endomembrane compartments leads to the colocalization of the promiscuous biotin ligase with its natural binding partners, the biotin ligase BirA, BccP, and the biotin operator, which may interfere with the detection of the proxisome of the protein of interest. One solution to this pitfall is to use biotin ligases from distantly related bacteria, as they are likely to have lower affinity for the orthologs of their binding partners. BioID2, derived from BirA of *Aquifex aeolicus*, meets this criterion, and its lack of a DNA binding domain, which it shares with other group 1 biotin ligases (41) and miniTurbo (32), is an advantage. Unfortunately, BioID2 and miniTurbo were neither well expressed nor very active in *Shigella*, which led us to consider BioID and TurboID instead. Our data indicate that TurboID binds less strongly to BccP than BioID. In addition, the binding of TurboID to the biotin operator is greatly reduced compared to BirA. This may be due to the increased instability of its complex with biotin-5′-AMP, the likely basis for its superior biotinylation promiscuity. Although the conditions found in the bacterial cytoplasm cannot be fully recapitulated in this *in vitro* assay, we believe that the data obtained support that TurboID is the best enzyme tested for studying bacterial proxisomes while reducing potential interference from the natural binding partners of BirA. However, it is important to emphasize that successful applications of miniTurbo have been reported in two other bacterial species belonging to the phylum *Pseudomonadota* (39, 40). We are not aware that promiscuous biotin ligases have been used in other phyla, nor are there any fundamental issues that would prevent their use.

On the other hand, the labeling volume of TurboID is significant compared to the volume of the *E. coli* cytoplasm. Assuming a spherical biotinylation cloud with a radius of 35 nm (34)—corresponding to a volume of 1.80 × 10^5^ nm^3^—and a cytosolic volume of 0.67 µm^3^ (BNID 100011) (45), a single molecule of TurboID would be able to label proteins occupying 2.7 × 10^−4^ of the cytoplasmic volume. Assuming there are 1,000 molecules of TurboID (≈1 µM) and that they are homogeneously distributed, 27% of the cytoplasm would be within a biotinylation cloud at any given time. Even using a more conservative labeling radius of 10 nm (32, 33), the clouds would still extend over 6% of the cytoplasm. Therefore, free diffusing TurboID would likely expose the entire cytoplasm to its biotinylation activity within seconds. In our data set, this is illustrated by the enrichment of a group of prestored T3SS substrates in the lysate of control cells expressing the free TurboID in the off-state. These observations underscore the critical importance of systematically comparing the enrichment of proteins obtained with the free TurboID and the TurboID fusions (46). This is based on the rationale that the diffusion pattern of free TurboID differs from that of TurboID fused to the protein of interest, which forms specific protein complexes as part of its normal cellular function. Critical to the success of this approach is having similar concentration of TurboID in the control and test samples to minimize the occurrence of false positives and false negatives and using SAINT (42) or equivalent to identify meaningful hits. The background noise of promiscuous biotin ligases in bacteria could probably be attenuated by several approaches (reviewed in reference 46), such as using biotin-depleted medium and reducing the cellular concentration of biotin ligases or their labeling time with weaker or inducible promoters.

**Fig 6:**
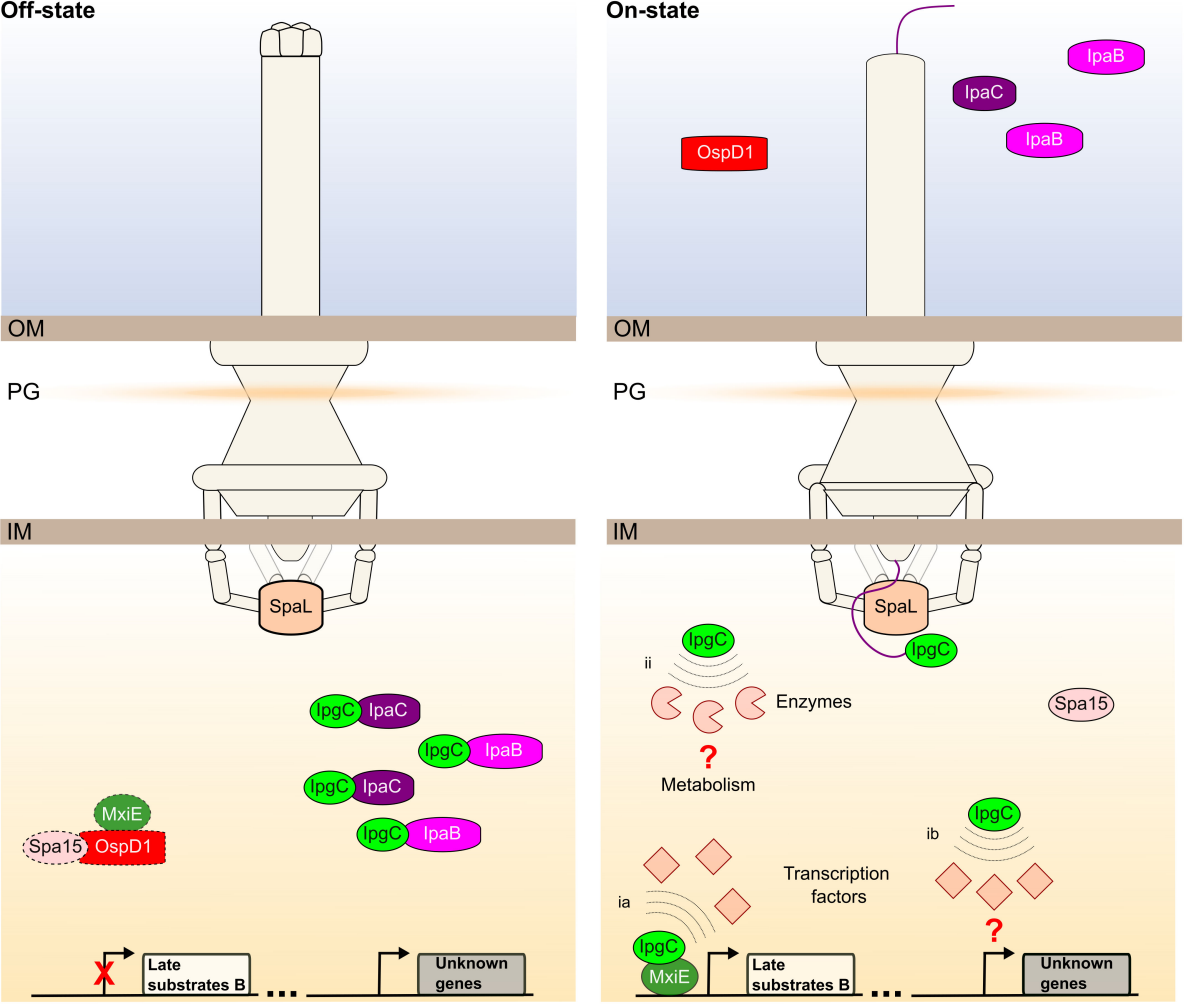
Model describing how the IpgC proxisome may be more complex when the T3SS is in the on-state. This schematic integrates insights from the IpgC proxisome into the seminal T3SS signaling model, which is as follows. In the off-state, MxiE and IpgC form inhibitory complexes with Spa15-OspD1 and IpaB or IpaC, respectively. In the on-state, IpgC escorts its cargo IpaBC to the T3SS sorting platform, which contains the ATPase SpaL (also known as Spa47 or SctN). This increases the cytoplasmic concentration of free IpgC, which can then interact with MxiE. Together, they form a transcriptional activation complex that is responsible for the expression of MxiE box-containing genes encoding T3SS late substrates B (e.g., IpaH). The proxisome revealed by TurboID supports the existence of IpgC-IpaBC and IpgC-MxiE complexes, while the MxiE-OspD1-Spa15 (dashed lines) could not be confirmed due to technical limitations. Furthermore, the proxisome data suggest that MxiE does not fully titrate IpgC molecules released by IpaBC secretion in the on-state. Therefore, IpgC diffuses to other areas of the cell where it colocalizes with transcription factors (ia-b) and metabolic enzymes (ii). IpgC could colocalize with transcription factors either through by binding to DNA in complex with MxiE (ia) or in a monomeric form associated or not with DNA (ib). The existence of cross-talk between the T3SS via IpgC and the functions of these transcription factors and enzymes is currently unknown. The T3SS signaling model has been described in reference 25, and this schematic is adapted from reference 12. OM: outer membrane; PG: peptidoglycan cell wall; IM: inner membrane.

The proxisome of IpgC revealed by TurboID included previously validated direct binding partners (Fig. 6). For example, IpaB and IpaC were enriched in the off-state, although they were also detected in the on-state. The latter is likely due to their transient interaction with IpgC, prior to secretion. In contrast, MxiE was enriched in the on-state, although less strongly than suggested by the T3SS signaling model. The data suggest that this is due to the high background biotinylation of MxiE by the free TurboID. Our data also revealed puzzling phenomena. For example, OspD1 is enriched in the on-state with MxiE as the bait, whereas the T3SS signaling model suggests that this interaction should be prominent in the off-state (25). Furthermore, OspD1 is enriched in the off-state with IpgC as the bait, whereas previous data suggested that they do not bind together (25). It would be premature to reconsider the T3SS signaling model based on these data. Indeed, these discrepancies are likely due to the limitations of proximity biotinylation. For the MxiE proxisome, the unexpected enrichment of the T3SS substrate OspD1 in the on-state may be due to the masking effect of its cytoplasmic accumulation in the off-state, leading to its higher background biotinylation by free TurboID in the latter state. In the case of the IpgC proxisome, it is notable that similarly to OspD1, other prestored T3SS substrates such as IpgD and IpaA are similarly enriched in the off-state with IpgC as the bait. Interestingly, IpaC, a cognate cargo of IpgC, was shown to localize at the cytoplasmic pole in the off-state (47, 48). Therefore, the vicinity of IpgC with T3SS substrates that are not among its cognate cargos could indicate that they are co-located in a specific region of the cytoplasm prior to their secretion. Alternatively, the higher cytoplasmic concentration of T3SS substrates in the off-state could lead to their artifactual identification. Taken together, these observations illustrate the challenge of using proximity biotinylation to study proteins whose cellular concentration varies significantly in the experimental conditions being compared.

Examination of the general behavior of IpgC suggests that it diffuses more freely within the cytoplasm in the on-state (Fig. 6). This conclusion is based on the observation that the number of proteins in the vicinity of IpgC is higher in this condition. It is consistent with the idea that the titration of IpgC by its cognate cargos IpaB and IpaC limits its diffusion in the off-state. Therefore, the decrease in cytoplasmic concentration of IpaB and IpaC due to their secretion in the on-state releases a significant amount of IpgC that is incompletely titrated by MxiE to form the MxiE-IpgC complex that binds the DNA. This would allow IpgC to localize in the vicinity of several other proteins, including components of the T3SS sorting platform such as SpaL (also known as Spa47 or SctN). IpgC may briefly interact with these components while escorting its cargo for secretion, as has been suggested for other T3SS chaperones (49–52). In addition, six transcription factors are found in the vicinity of IpgC. It is plausible that DNA could link IpgC or IpgC-MxiE to this select group of transcription factors. Indeed, some of them have been associated with IpgC or with the T3SS. For example, a negative feedback loop targeting VirF and involving IpgC and MxiE has been proposed to repress T3SS genes by dampening the expression of the transcription factor VirB (53). This process may lead to the colocalization of IpgC with VirF. In contrast, with VirF and MxiE that belong to the AraC/XylS family, unrelated chromosome encoded transcription factors McbR, SlyA, ModE, KdgR, and CysB were also enriched in the vicinity of IpgC when the T3SS was actively secreting. KdgR and CysB have been implicated in the regulation of the T3SS in other bacteria (54, 55). McbR and SlyA coregulate the expression of the ribosome modulation factor and of GadX, which are both important for the adaptation to stressful conditions (56). In addition, the overexpression of SlyA can activate *Shigella* T3SS expression under conditions that are otherwise not permissive for virulence (57). Therefore, the connections between these transcription factors and the T3SS warrant further experimental validations. Several housekeeping proteins were also enriched around IpgC in the on-state. Indeed, a subset including AtpG, GcvP, GdhA, GltA, Pgk, Ppc, and PurF form a highly connected network according to STRING. Knockout of *gltA* was reported to increase the expression of T3SS genes in *Pseudomonas aeruginosa* through activation of the stringent response (58). Disruption of the synthesis of the stringent response alarmone (p)ppGpp reduced the expression of T3SS genes in *Shigella* (59), but the role of *gltA* in this process is unknown. However, it is unclear what their relationship might be, as IpgC is not known to interact with housekeeping proteins.

This study has limitations that need to be considered. For example, the on-state is mimicked with strains that have a knockout in one of the two genes encoding the tip complex proteins. Secretion in these strains is constitutive and is maintained for a long period of time, dwarfing that of infection, which could cause changes in bacterial physiology. Using free TurboID in the same strain as a control for the proxisome study mitigates this problem. Furthermore, the alternative of using the chemical T3SS inducer Congo red is not satisfactory as it also affects cell growth and fitness. Second, we used a mild detergent for cell lysis, which may have adversely affected the isolation of hydrophobic proteins such as the components of the T3SS that are integral to the plasma membrane. Third, it is important to consider that TurboID is a relatively large enzyme (~35 kDa) which fusion with smaller proteins like IpgC (~18 kDa) and MxiE (~25 kDa) may affect their diffusion and capacity to form molecular complexes. We only partially addressed this by studying both N- and C-terminal protein fusions. Finally, we did not use experimental conditions that prevented the accumulation of biotinylated proteins prior to the addition of supplemental biotin. Therefore, a significant number of biotinylated proteins had accumulated prior to the biotin pulse.

In conclusion, this study describes one of the first applications of the promiscuous biotin ligase TurboID in the bacterial cytoplasm. It has revealed putative novel cross-talks of the *Shigella* T3SS chaperone IpgC with cellular processes, some of which have been associated with the T3SS in other bacteria. Future exciting developments include the study of these cross-talks and, of course, the pursuit of method development for proximity labeling in prokaryotes.

## MATERIALS AND METHODS

### Bacterial strains

We used *Shigella flexneri* serotype 5a strain M90T with acquired streptomycin resistance as the WT strain (60). Its isogenic mutant strains Δ*ipaD,* Δ*mxiE*, *ipaB4*, and *ipaB4* Δ*mxiE* were a kind gift from Philippe Sansonetti and Claude Parsot (15, 23) (see Table S5 for strains description). *S. flexneri* was grown on tryptic soy agar (TSA) and tryptic soy broth (TSB). *E. coli* BL21 Rosetta strain (Novagen), a kind gift of Roberto Chica, was grown on Luria-Bertani agar or broth. Media were supplemented with 100 µg/mL ampicillin, 30 µg/mL chloramphenicol, 12.5 µg/mL zeocin, 50 µg/mL gentamicin, or 50 µg/mL kanamycin as appropriate for plasmid selection and maintenance or to confirm the genotype of mutant strains. Δ*ipgC* and Δ*ipaD* Δ*ipgC* were generated by allelic exchange (61), in the WT and isogenic Δ*ipaD* strains described above, and inspired by the genetic makeup of the original nonpolar Δ*ipgC* mutant (26). In brief, the zeocin (*zeo*) resistance cassette was inserted between base pair 128 and 453 of *ipgC*, leaving 42 codons and 5 codons upstream and downstream of the *zeo* resistance cassette, respectively. The *zeo* amplicon was obtained with primers GMO28/29 using the template pZeo/SV2+ (see Table S6 for primers list). Mutant clones were isolated on TSA supplemented with 12.5 µg/mL zeocin and 0.01% Congo red (Fisher Scientific). The correct insertion of zeo was confirmed by colony screening PCR using primers GMO24/27 and GMO25/26 for the 5´ and 3´ junction, respectively.

### Plasmids

The coding sequence of *bioID*, *bioID2*, *turboID*, and *miniTurbo* [Addgene plasmids #35700 and #74224, kind gift from Kyle Roux (30, 31); #107169 and #107170, kind gift from Alice Ting (32)] were amplified by PCR using primers HMIO 607/609, 11/14, 249/251, and 247/251, respectively, which inserted EcoRI (5´-end), and XbaI restriction sites and a Myc tag (3´-end). The resulting amplicons were digested with EcoRI and XbaI and then subcloned by ligation into a similarly digested pSU2.1 (62), a derivative of pSU2718 (GenBank: M64731.1), yielding pSU2.1 *bioID* (pNHA1), *bioID2* (pNHA2), *turboID* (pNHA3), or *miniTurbo* (pNHA4). These pSU2.1 derivatives possess a *lac* promoter that is constitutively expressed due to the absence of the lacI on the plasmid. To construct the plasmids needed for protein purification, *turboID* and *BioID* were amplified using HMIO408/409 and HMIO404/405 and ligated into BamHI and KpnI of pQE-80L (Qiagen) to yield pQE-80L *turboID* (pNHA5) and pQE-80L *bioID* (pNHA6). To obtain pQE-80L *birA* (pNHA7), a G118R mutation was introduced into pNHA6 by mutagenesis PCR with primers HMIO650/651 (29). Finally, *bccP* was cloned from *Shigella flexneri* M90T by PCR using primers HMIO402/403 and inserted into pQE-80L *MBP-icaR* in place of *icaR* [Silué et Campbell-Valois, unpublished, and reference (62)], yielding pQE-80L *MBP-bccP* (pNHA8), using BamHI and PstI restriction sites. To obtain pSU2.1 *ipgC-turboID-myc*, the *ipgC* amplicon was obtained with primers FXO179/180, digested with BglII and BamHI and ligated through BamHI into pSU2.1; the *turboID-myc* amplicon was then obtained with primers HMIO250/251 and ligated 3´ of *ipgC* into BamH-XbaI restriction sites. To yield a satisfactory level of expression, *turboID-myc* and *ipgC-turboID-myc* were then subcloned through XhoI and XbaI overhangs into pUC18.1rp (pNHA9), a derivative of pUC18 with the constitutive *rpsM* promoter, yielding pUC18.1rp *turboID-myc* (pNHA10), and pUC18.1rp *ipgC-turboID-myc* (pNHA11). The MxiE amplicon obtained with HMIO699/701 was swapped with *ipgC* from pUC18.1rp *ipgC-turboID-myc* through ligation of EcoRI and BamHI digested fragments to yield pUC18.1rp *mxiE-turboID-myc* (pNHA13). Finally, the *turboID-myc-ipgC* and *turboID-myc-mxiE* were derived in two steps from pUC18.1rp *turboID-myc*. First, the entry plasmid was obtained by introducing KpnI, SacI, and BamHI restriction sites downstream of *myc* in pUC18.1rp *turboID-myc* by mutagenesis PCR with primers HMIO694/695. Second, *mxiE* and *ipgC* amplicons generated with primers HMIO698/699 and HMIO734/735 were ligated into this entry plasmid using KpnI and BamHI and KpnI and SacI, respectively, yielding pUC18.1rp *turboID-myc-ipgC* (pNHA12) and pUC18.1rp *turboID-myc-mxiE* (pNHA14). Notably, IpgC/MxiE-TurboID-Myc and TurboID-Myc-IpgC/MxiE protein fusions have a linker between the TurboID and the bait proteins that measures 5 amino acids (GSGGG) and 14 amino acids (SGEQKLISEEDLGT; the Myc tag is underlined), respectively. Therefore, both N-terminal and C-terminal TurboID fusions with IpgC or MxiE have the same molecular weights, i.e. 54 kDa and 66 kDa, respectively. Phusion High-Fidelity DNA polymerase was used to generate all amplicons used for cloning (Thermo Scientific, #F530) and restriction enzymes were purchased from Thermo Scientific. All constructs were verified by Sanger sequencing (Génome Québec). For practical reasons, the main plasmids described above were given short names that are used in the rest of the methods (see Table S7 for plasmids list).

### Comparison of the different promiscuous biotin ligases by immunoblotting

*Shigella flexneri* strain M90T WT harboring either pSU2.1, pNHA1, 2, 3, or 4 (Table S7) were grown overnight in TSB supplemented with the corresponding antibiotics at 30°C with shaking at 250 rpm. The next day, the overnight cultures were subcultured 1/50 in fresh TSB supplemented with half the regular concentration of antibiotics and incubated at 37°C until they reach an optical density at 600 nm in a 1 cm light path (OD600) ≈ 1. Then, 50 µM biotin (Sigma-Aldrich, B4501) was added to each tube, and they were incubated at 37°C for the indicated time. The volume of cell culture from each strain was adjusted based on the OD600, pelleted, resuspended in Laemmli buffer (1×), and heated for 10 min at 95°C. Samples were run on 12% SDS-PAGE gels and then transferred to a nitrocellulose membrane (Bio-Rad, 1620115) to measure biotinylation of the cell lysate. The membrane was blocked in phosphate-buffered saline (PBS) with 1% bovin serum albumin (BSA) and 0.2% TritonX for 10 min then incubated with streptavidin-horseradish peroxidase conjugate (Cytiva, RPN1231) for 40 min at room temperature followed by three washes of 10 min in PBS. Notably, the endogenous biotinylated protein BccP (MW 17 kDa) masked the signal of the lower active BioID2. This phenomenon is not problematic with the highly active BioID and TurboID. Nevertheless, we generally avoided imaging biotinylated protein with MW <20 kDa by adjusting the migration time to elute them from the gel or by discarding this section of the membrane. To evaluate protein expression, the same samples were run on separate gels and then transferred to a polyvinylidene difluoride (PVDF) membrane (Bio-Rad, 1620177), and immunoblotting was performed using 1/10,000 mouse anti-Myc (Genescript, A00704) as the primary antibody, and 1/20,000 anti-mouse IgG-HRP (Jackson Immunoresearch, 115-035-003) as the secondary antibody. Both nitrocellulose and PVDF membranes were soaked in Clarity Western ECL substrate (Bio-Rad, 170-5061), and images were acquired using a ChemiDoc XRS+ system (Bio-Rad). The activity of the four biotin ligases was assessed by quantification of biotinylated proteins at 10 min, measuring pixel intensities in the range from 28 to 150 kDa for each lane (*n* = 3) with ImageJ (version 1.54f). To better quantify the expression and activity of TurboID and miniTurbo, three replicates were repeated on the same membrane and activity was evaluated while considering the expression level of the two biotin ligases and error propagation. Graphs were plotted using GraphPad Prism showing data as mean ± standard deviation. Comparisons of multiple samples were performed using one-way analysis of variance (ANOVA) followed by Tukey’s test. Comparisons between TurboID and miniTurbo were performed using unpaired Student’s *t*-test.

### Purification of biotin ligases and BccP

pQE-80L-derived plasmids pNHA5-7 harboring 6×His-biotin ligase and the plasmid pNHA8 harboring *MBP-bccP* were introduced into BL21 Rosetta *E. coli* strain. Cultures were grown in terrific broth (TB) and 2xYT for the biotin ligases and *bccP*, respectively, both supplemented with ampicillin. Protein expression was induced with 1 mM isopropyl-β-D-thiogalactoside at OD600 ≈ 0.6 and incubated for 24 hours at 16°C. The cells were then collected by centrifugation and resuspended in lysis buffer (50 mM sodium phosphate, 300 mM NaCl, 10 mM imidazole, 1 mM phenylmethylsulfonyl fluoride [PMSF], 150 µg/mL lysozyme [BioShop, LYS702], 10 µg/mL DNase I (Sigma-Aldrich DN25), 10 mM MgCl_2,_ pH 7.4) for biotin ligases or (50 mM tris-HCl, 150 mM NaCl, 1 mM PMSF, 150 µg/mL lysozyme, 10 µg/mL DNase I, 10 mM MgCl_2_, pH 8.0) for BccP. Cells were sonicated and clarified by centrifugation. The soluble cell lysate was incubated with HisPur resin (Thermo Scientific, 88221) for the Histag or amylose resin (New England Biolabs, E8021S) for the MBP at room temperature for 1 hour with end-over-end rotation and then transferred into Pierce Centrifuge Columns (Thermo Scientific, 89868). HisPur resin was then washed with 50 mM sodium phosphate, 300 mM NaCl, 25 mM imidazole, pH 7.4, and proteins were eluted with 50 mM sodium phosphate, 300 mM NaCl, 250 mM imidazole, pH 7.4, and desalted using Zeba Spin Desalting Columns (Thermo Scientific, 89890) in a storage buffer (40 mM Tris-HCl [pH 8.0], 100 mM KCl, 10% glycerol). The same procedure was used with the amylose resin, except the wash (20 mM Tris-HCl [pH 7.4], 200 mM NaCl, 1 mM EDTA) and elution buffer (20 mM Tris-HCl [pH 7.4], 200 mM NaCl, 1 mM EDTA, 10 mM maltose) were modified as indicated. Protein samples were frozen in an ethanol dry ice bath and then stored at −80°C until further use. The use of 2× YT medium instead of TB was crucial to obtain a significant fraction of purified BccP in its unbiotinylated form. However, an additional purification step on streptavidin-agarose beads (Thermo Scientific, 20353) was required to remove biotinylated BccP. Briefly, the eluate from the amylose resin was incubated at room temperature with streptavidin-agarose using an end-over-end rotator to capture holo BccP (biotinylated). The beads were pelleted by centrifugation and the resulting apo BccP-enriched supernatant was used to perform the biochemical assays described below.

### *In vitro* biotinylation assay to compare BccP binding activity of BioID and TurboID

This assay was performed as previously described (29) by incubating 20 nM of the purified biotin ligases with 2 µM BccP or RNase A (Sigma-Aldrich, R6513) for different time points ranging from 3 min to 24 hours at 37°C in a reaction buffer (40 mM Tris-HCl [pH 8.0], 3 mM ATP, 5.5 mM MgCl_2_, 100 mM KCl, 1.4 mM β-mercaptoethanol, and 5 µM biotin). The reaction was stopped by adding Laemmli buffer and heating at 95°C for 10 min. Samples were run on a 12% SDS-PAGE gel then analyzed by Western blotting with streptavidin-horseradish peroxidase conjugate. Band intensities (*n* = 3) were measured using ImageJ, as described above, and timecourse graphs were plotted and fitted using KaleidaGraph version 5.0 (Synergy Software), using the equation for the level of biotinylation *B* in function of time, *t*:


$$B(t)=\frac{B^{I}*t}{t^{50}+t}$$

where *B^I^* is the level of biotinylation when *t→∞*, and *t*^50^ is the midpoint of biotinylation, i.e., the time at which 50% of the protein is biotinylated.

### Electrophoretic mobility shift assay

The 112 bp biotin operator was obtained from *Shigella flexneri* strain M90T by PCR with primers HMIO702–703 described in reference (63). The PCR product was run on a 2% polyacrylamide gel and purified with a gel and PCR purification kit to obtain the biotin operator DNA probe. Gel shift assay was performed by incubating for 30 min at room temperature, 40 nM of this DNA probe with different concentrations (50 nM, 150 nM, 250 nM) of each biotin ligase tested, in a reaction buffer (50 mM tris-HCl pH 8.0, 1 mM EDTA, 50 mM NaCl, 1 mM ATP, 1 mM MgCl_2_, 1 µM biotin, 10% glycerol). A 5% polyacrylamide retardation gel was prerun in 0.5× tris-acetate-EDTA buffer (TAE) at room temperature for 30 min (100V) before the reaction mixtures were loaded onto it and run at 4°C for 45 min (100V). The gels were washed with ddH_2_O and stained with 1 µg/mL ethidium bromide for 20 min then visualized using a ChemiDoc XRS+ system. A Coomassie-stained SDS gel was prepared in parallel as a loading control for the samples. Band intensities (*n* = 3) were measured using ImageJ, as described above, and graphs were plotted using GraphPad Prism showing data as mean ± standard deviation. DNA shift in each sample was compared to the free DNA using one-way ANOVA followed by Dunnett’s multiple comparisons test.

### Complementation and biotinylation assay for the TurboID fusions with IpgC and MxiE

The strains Δ*ipgC* and Δ*ipaD* Δ*ipgC* harboring pUC18.1rp-derived plasmids pNHA10, 11, and 12 and strains Δ*mxiE* and *ipaB4* Δ*mxiE* harboring pUC18.1rp-derived plasmids pNHA10, 13, and 14 were used to test for the complementation. *Shigella flexneri* Str M90T WT, Δ*ipaD*, and *ipaB4* harboring pNHA9 were used as the reference for the off-state and on-state of the T3SS. These strains were incubated overnight at 30 degrees with shaking at 250 rpm in TSB supplemented with ampicillin. The next day, a 1/50 subculture was prepared in TSB and incubated at 37°C until an OD600 ≈ 1 was reached. Cells were quantified based on their OD, pelleted, resuspended in 1× Laemmli buffer, and heated for 10 min at 95°C. Samples were run on a 12% SDS-PAGE gel then analyzed by Western blot as described above using the mouse anti-Myc primary antibodies, 1/10,000 rabbit polyclonal anti-IpgC (26), and 1/3,000 rabbit anti-ipaH (23). The biotinylation assay was performed as described above with minor modifications. Bacterial cultures were then supplemented with 50 µM biotin, except for the no supplemental biotin control, and further incubated at 37°C for 10 and 20 min. Protein samples were prepared and analyzed by Western blot as described previously using streptavidin-horseradish peroxidase conjugate, mouse anti-Myc and 1/1,000 anti-RecA (MBL, ARM191) primary antibodies, and anti-mouse IgG-HRP and anti-rabbit IgG-HRP secondary antibodies (Jackson Immunoresearch, 111-035-003). Quantification of biotinylated proteins by each fusion in different time points was assessed in each strain for the IpgC-TurboID fusions, by measuring pixel intensities in the range from 28 to 150 kDa for each lane (*n* = 3) with ImageJ, as described above. To calculate the percentage of biotinylation events that occurred in the 10 min of incubation with the supplemental biotin, we subtracted relative pixel intensities in the no supplemental biotin control (%) from pixel intensities in the 10 min with supplemental biotin (%) for each strain discussed.

### Preparation of proteins for mass spectrometry analyses

The strains described in the complementation experiment were grown overnight at 30°C in TSB supplemented with ampicillin. The next day, a 1/50 dilution was prepared in 30 mL TSB supplemented with 50 mg/mL of ampicillin and incubated at 37°C until an OD600 ≈ 1. Next, 50 µM biotin was added to each flask, which were then incubated at 37°C for 10 min. Samples were cooled on ice for 15 min and washed with buffer W (50 mM HEPES [Fisher Scientific, BP310; pH 7.3], 200 mM NaCl, 10% glycerol, 1 mM PMSF [Sigma-Aldrich P7626], and 2 mM EDTA), then resuspended in 4 mL of lysis buffer (buffer W supplemented with 1% [wt/vol] n-dodecyl-β-D-maltoside [Cayman Chemical Company, 16494]). Cells were lysed by sonication with 30 s pulses (0.85 cycle, 80% amplitude) repeated four times with a 30 s rest on ice between pulses. Purification of biotinylated proteins and collection of tryptic peptides were adapted from reference 64. Samples were centrifuged at 17,000 × *g* at 4°C for 20 min, and the resulting supernatant was mixed with 50 µL of PBS-washed streptavidin-agarose beads (Thermo Scientific, 20353) by end-over-end rotation at room temperature for 1.5 hours. The beads were then collected by centrifugation at 2,000 × *g* for 2 min and washed three times with 1% SDS using Bio-Spin Columns (Bio-Rad, 7326204), followed by three washes with 6 M urea (Bio-Rad, 161-0731). Protein-loaded streptavidin beads were washed one time with PBS and five times with 50 mM ammonium bicarbonate or ABC (Thermo Scientific, 393212500) in Bio-Spin Columns then transferred to microcentrifuge tubes and pelleted at 2,000 × *g* for 2 min. The beads were resuspended in 10 mM DTT (Thermo Scientific, R0861) in ABC and heated for 30 min at 56°C followed by the addition of iodoacetamide (Thermo Scientific, 122270050) at a final concentration of 25 mM and an incubation in the dark with rotation at room temperature for 30 min. The beads were washed in ABC supplemented with 10 mM DTT for 5 min followed by three washes with 50 mM triethylammonium bicarbonate or TEAB (Thermo Scientific, 90114), pH 8.5. The proteins were then digested off the bead with 10 µg/mL trypsin (Promega, V5113) in TEAB while shaking overnight at 37°C. Digested peptides were recovered by centrifugation and filtration using Bio-Spin Columns. The samples were dried using vacuum centrifugation at 1,500 rpm and room temperature.

### Mass spectrometry-based proteomics analyses

The samples were analyzed at the John L. Holmes Mass Spectrometry Facility (Faculty of Science, University of Ottawa), as previously described (65). Briefly, the tryptic peptides obtained in the previous section were analyzed by an Orbitrap Fusion mass spectrometer (Thermo Scientific) coupled to a Dionex UltiMate 3000 RSLCnano system (Thermo Scientific) with an in-house-packed 70 µm ID × 150 mm length separation column (Polymicro Technology), Luna C18(2), 3 µm, 100 Å (Phenomenex). Samples were injected and separated through a gradient solution of 0.1% formic acid and acetonitrile in deionized water. They were applied to the column for a total of 105 min at a flow rate of 0.30 µL/min, which divided as follows. The peptides were separated with 2% acetonitrile for the first 7 min, then the gradient was linearly increased to 38% for the next 70 min, then from 38 to 98% for 5 min, remained at 98% for 10 min, then gradually decreased from 98 to 2% acetonitrile for 3 min, and finally washed with 2% acetonitrile for 10 min. Peptides were ionized using nano-electrospray ionization (ESI) at an ion source temperature of 250°C with an ion spray voltage of 2.1 kV. Survey scans between 300 and 2,000 *m*/*z* were acquired at 60K resolution, and precursors with charge state +2 to +7 were filtered according to the monoisotopic precursor selection. The dynamic exclusion time was set to 30 s with a tolerance of 10 ppm. Automatic gain control settings were set to 5 × 10^5^ for full Fourier transform mass spectrometry (FTMS) scans and 1 × 10^4^ for tandem mass spectrometry (MS/MS) scans. Precursors were isolated using a 2 *m/z* isolation frame, and collision-induced dissociation fragmentation was performed with a collision energy of 35%.

### Mass spectrometry computational data analysis

The raw mass spectrometry data were processed using the Trans-Proteomic Pipeline (v6.3.0 Arcus, Build 202305021110-8944 for WindowsNT-x8664) (66), as follows. The raw files were converted to mzML using ProteoWizard’s msconvert (version 3.0.22340) (67). Comet (release 2023.01 rev.2) (68) was used for peptide-spectrum matching against a protein sequence database made of the proteome of *S. flexneri* 5a strain M90T (NZ_CP037923.1) and its virulence plasmid pWR100 (NC_024996.1) from the NCBI RefSeq database along with the “common contaminants” from the Max Planck Institute and their reversed counterpart for a target-decoy database search (10,269 sequences total). Semi-tryptic peptides with at most three miscleavages were searched by Comet with a precursor mass tolerance of 20 ppm. Carbamidomethylation (C) was considered as a static modification and oxidation (M) and deamidation (NQ) were considered as variable modifications. Confidence of Comet peptide-spectrum match results was assessed using PeptideProphet (69), ProteinProphet, and iProphet (70, 71). Label-free quantification was achieved using StPeter (version 1.4.0) (72) with 1% FDR cutoffs and a probability threshold associated with a calculated 1% FDR for the input data, protein samples were set at 20 µg, and degenerate peptide quantification was allowed. The protein lists were exported to include only the sequences under the calculated 1% FDR threshold in each replicate with the DECOY and contaminant sequences filtered out.

### Computational assessment of proxisome

The SAINT software package (version 2.5.0) (42) was used to calculate the probability of the detected proteins being enriched in the test versus control samples using spectral counts. The test samples consisted of five (IpgC) and six (MxiE) biological replicates for each TurboID fusion (N- or C-terminal). The control samples consisted of two or three biological replicates of the respective knockout strains used to mimic the on-state and off-state of the T3SS (Δ*ipgC*, Δ*ipaD* Δ*ipgC*, Δ*mxiE*, and *ipaB4* Δ*mxiE*) expressing the free TurboID, and of non-TurboID expressing strains (M90T WT and Δ*ipaD*). For each bait, two SAINT analyses were run corresponding to the off- and on-state, each including both N- and C-terminal TurboID fusions as independent test groups. SAINT was run with recommended settings. Ten percent FDR cutoffs including, at a minimum, all interactions with a probability >90% was used to identify confident vicinal proteins. The FC of a protein p was calculated using LFQ intensities of p with this formula:


FC=[LFQ(p) test on/LFQ(p) control on]/[LFQ(p) test off/LFQ(p) control off]

in which “on” and “off” indicate the LFQ in the on-state and off-state, respectively, tests were either the N-terminal or C-terminal fusions of TurboID with IpgC or MxiE and the controls were the corresponding free TurboID, as described above. Proteins that were undetected in one or more of the samples (LFQ = 0) were imputed with LFQ = 1 to perform the FC calculation. To plot these data, FC values for proteins FC < 1 (i.e., those enriched in the off-state) were inverted to obtain FC > 1, and proteins enriched in the off-state and on-state were identified with red and blue dots, respectively.

### STRING network

A STRING network of selected proteins found in the proxisome of IpgC according to TurboID was generated using STRING v.12.0 taking into consideration known interactions that were experimentally determined or detected in curated databases, as well as predicted interactions based on gene neighboring or co-occurrence, and co-expression. An FDR stringency of 1% and a medium confidence of 0.400 were applied using the *E. coli* K-12 database. All proteins showed in Fig. 5C and conserved in *E. coli* K-12 were used to generate the initial network. The most highly interconnected region of this network is shown in Fig. 5D. The GO term analyses were performed on this subnetwork with the whole *E. coli* K-12 genome with threshold FDR ≤ 5% and strength >0.01.

## Data Availability

The mass spectrometry data are available from the Mass Spectrometry Interactive Virtual Environment (MassIVE) at ftp://massive.ucsd.edu/v08/MSV000095473/.

## SUPPLEMENTAL MATERIAL

Supplemental Tables msphere.00553-24-s0001.docx Table S5 to S7.

Table S1 msphere.00553-24-s0002.xlsx Raw mass spectrometry data and SAINT analyses for IpgC.

Table S2 msphere.00553-24-s0003.xlsx Raw mass spectrometry data and SAINT analyses for MxiE.

Table S3 msphere.00553-24-s0004.xlsx Fold change of proteins found in the proxisome of IpgC according to SAINT analyses.

Table S4 msphere.00553-24-s0005.xlsx Fold change of proteins found in the proxisome of MxiE according to SAINT analyses.

ASM does not own the copyrights to Supplemental Material that may be linked to, or accessed through, an article. The authors have granted ASM a non-exclusive, world-wide license to publish the Supplemental Material files. Please contact the corresponding author directly for reuse.
