# Supplementary material for: The promiscuous biotin ligase TurboID reveals the proxisome of the T3SS chaperone IpgC in Shigella flexneri
Source: mSphere. 2024 Oct 31;9(11):e00553-24. doi: 10.1128/msphere.00553-24 (PMC11580435; doi:10.1128/msphere.00553-24)
Supplement: Supplemental Tables — Table S5 to S7. [file msphere.00553-24-s0001.docx]

**Supplemental Materials for:**

**The promiscuous biotin ligase TurboID reveals the proxisome of the T3SS chaperone IpgC in *Shigella flexneri***

Nathaline Haidar-Ahmad^a,b^, Kyle Tomaro^a,b,c^, Mathieu Lavallée-Adam^c^, François-Xavier Campbell-Valois^a,b^#

^a^ Department of Chemistry and Biomolecular Sciences, Centre for Chemical and Synthetic Biology, Host-Microbe Interactions Laboratory, University of Ottawa, Ottawa, ON, Canada

^b^ Department of Biochemistry, Microbiology and Immunology, Centre for Infection, Immunity and Inflammation, University of Ottawa, Ottawa, ON, Canada

^c^ Department of Biochemistry, Microbiology and Immunology. Ottawa Institute of Systems Biology, University of Ottawa, Ottawa, ON, Canada

**Running Title: TurboID reveals the proxisome of IpgC**

#Address correspondence to François-Xavier Campbell-Valois, [fcampbel@uottawa.ca](mailto:fcampbel@uottawa.ca)

**Supplemental Tables**

**Table S1-S4:** Mass spectrometry data; see excel files separately attached with the manuscript.

**Table S5.** *Shigella* strains used in this study

| **Bacterial Strain** | **Description** | **Antimicrobial resistance** | **Reference** |
| --- | --- | --- | --- |
| *S. flexneri* M90T (WT) | *Shigella flexneri* serotype 5a strain M90T with a spontaneous streptomycin resistant mutation | streptomycin | (1) |
| *S. flexneri* Δ*ipaD* | Derivative of WT M90T with an *aphA3* cassette  inserted after codon 130 of *ipaD* | kanamycin | (2) |
| *S. flexneri* Δ*ipgC* | Derivative of WT M90T with a zeocin (*zeo*) resistance cassette inserted after codon 42 of *ipgC.* | zeocin | This study |
| *S. flexneri* Δ*ipaD* Δ*ipgC* | Derivative of Δ*ipaD* with a Zeocin (*zeo*) resistance cassette inserted after codon 42 of *ipgC.* | kanamycin & zeocin | This study |
| *S. flexneri* Δ*mxiE* | Derivative of WT M90T with an *aphA3* cassette  inserted after codon 134 of *mxiE* | kanamycin | (3) |
| *S. flexneri* *ipaB4* | Derivative of WT M90T with a gentamicin resistance cassette inserted after codon 337 of *ipaB* | gentamicin | (3) |
| *S. flexneri* *ipaB4* Δ*mxiE* | Derivative of Δ*mxiE* with a gentamicin resistance cassette inserted after codon 337 of *ipaB* | kanamycin &  gentamicin | (3) |

**Table S6.** Primers used in this study

| **Primer** | **Sequence** | **Description** |
| --- | --- | --- |
| GMO24 | CAGGCCAGGGTGTTGTCC | Check 5’ end of *ipgC::zeo*; Reverse primer binding around the 60^th^ codon of *zeo*; used with GMO27 |
| GMO25 | GGACAACACCCTGGCCTG | Check 3’ end of *ipgC::zeo*; forward primer binding around the 66^th^ codon of *zeo*; used with GMO26 |
| GMO26 | ATTTGCAGCTTGGATAGTATTGT | Check 3’ end of *ipgC::zeo*; forward primer binding around the 23^rd^ codon of *ipaB*; used with GMO25 |
| GMO27 | ACCGAAAATGAAAGCATCTCTA | Check 5’ end of *ipgC::zeo*; Forward primer binding around the 12^th^ codon of *ipgC*; used with GMO24 |
| GMO28 | GCAATTCCTGATGATATGATGGATGACATTTATTCATATGTGACTAACTAGGAGGAATAAATGAAAGCCAAGTTGACCAGT | Allelic exchange; binds 5’ end of *ze*o and add *ipgC* homology arm |
| GMO29 | GTGGTGCTTACATTATGCATAATAATTACTCCTTGATATCTCAGTCCTGCTCCTC | Allelic exchange; binds 3’ end of *zeo* and add *ipgC* homology arm |
| HMIO11 | AGAGGAATTCAGAGGGTATTAATAATGAAAGAACAGAAGCTTATTAGCGAAGAGGATCTGTCCGGATTCAAGAACCTGATCTGGCTGA | Cloning; binds 5’ end of *bioID2* and adds EcoRI and ribosomal binding site |
| HMIO14 | AGAGTCTAGATTAGTTACAGATCCTCTTCGCTAATAAGCTTCTGTTCTCCGGAGCTTCTTCTCAGGCTGAACTC | Cloning; binds 3’ end of *bioID2* and adds Myc tag |
| HMIO247 | AGAGGAATTCTAGGAGGTGCTAGCGCTATGATCCCGCTGCTGAACGC | Cloning; binds 5’ end of *miniTurbo* and adds EcoRI and ribosomal binding site |
| HMIO248 | AGAGGGATCCGGCGGTGGCATCCCGCTGCTGAACGC | Cloning; binds 5’ end of *miniTurbo* and adds BamHI and 5 AA linker |
| HMIO249 | AGAGGAATTCTAGGAGGTGCTAGCGCTATGAAAGACAATACTGTGCCTCTGAAGC | Cloning; binds 5’ end of *turboID* and adds EcoRI and ribosomal binding site |
| FXO179 | AGAGAGAGATCTTTAAATATCACCGAAAATGAAA | Cloning; binds 5’ end of *ipgC* and adds BglII |
| FXO180 | AGAGAGGGATCCCTCCTTGATATCCTGAATTGC | Cloning; binds 3’ end of *ipgC* and adds BamHI |
| HMIO250 | AGAGGGATCCGGCGGTGGCAAAGACAATACTGTGCCTCTGAAGC | Cloning; binds 5’ end of *turboID* and adds EcoRI and ribosomal binding site |
| HMIO251 | AGAGTCTAGATTAGTTACAGATCCTCTTCGCTAATAAGCTTCTGTTCTCCGGACTTTTCGGCAGACCGCAGA | Cloning; binds 3’end of *turboID* and miniTurbo and adds XbaI and Myc tag (14 AA addition) |
| HMIO402 | AGAGGGATCCGAAATCAGTGGTCACATCGTACG | Cloning; binds 5’end of *bccP* and adds BamHI |
| HMIO403 | AGAGCTGCAGTTACTCGATGACGACCAGCGG | Cloning; binds 3’end of *bccP* and adds PstI |
| HMIO404 | AGAGGGATCCAAGGACAACACCGTGCCCCT | Cloning; binds 5’end of *bioID* and adds BamHI |
| HMIO405 | AGAGGGTACCTTACTTCTCTGCGCTTCTCAGGGAGAT | Cloning; binds 3’end of *bioID* and adds KpnI |
| HMIO408 | AGAGGGATCCAAAGACAATACTGTGCCTCTGAAG | Cloning; binds 5’end of *turboID* and adds BamHI |
| HMIO409 | AGAGGGTACCTTACTTTTCGGCAGACCGCAG | Cloning; binds 3’end of *turboID* and adds KpnI |
| HMIO607 | AGAGGAATTCTAGGAGGTGCTAGCGCTATGAAGGACAACACCGTGCCCCT | Cloning; binds 5’ end of *bioID* and adds EcoRI and ribosomal binding site |
| HMIO609 | AGAGTCTAGATTAGTTACAGATCCTCTTCGCTAATAAGCTTCTGTTCTCCGGACTTCTCTGCGCTTCTCAGGGAGAT | Cloning; binds 3’end of *bioID* and adds XbaI and Myc tag (14 AA addition) |
| HMIO650 | CGCAGAGGACGGAAGTGGT | Mutagenesis; forward primer to introduce G118R to revert *bioID* to *birA* |
| HMIO651 | TCCTCTGCCAGCCTGCTG | Mutagenesis; reverse primer to introduce G118R to revert *bioID* to *birA* |
| HMIO694 | CTCGGTACCCAGATCCTCTTCGCTAATAAGC | Mutagenesis; forward primer to introduce KpnI-SacI-BamHI at the 3’ of the Myc tag in pUC18.1rp*TurboID-Myc* |
| HMIO695 | CTCGGATCCTAACTAATCTAGAGTCGACCTGC | Mutagenesis; reverser primer to introduce KpnI-SacI-BamHI at the 3’ of the Myc tag in pUC18.1rp*TurboID-Myc* |
| HMIO698 | AGAGAGGGTACCATGGGATCAAGTAAATATAAAGGTCTAAATACAAGTA | Cloning; binds 5’end of *mxiE* and adds KpnI |
| HMIO699 | AGAGAGGGATCCCTCGAGCCCAATTTTTTCATTTATTTTTTTCA | Cloning; binds 3’end of *mxiE* and adds BamHI |
| HMIO701 | AGAGAGGAATTCATGGGATCAAGTAAATATAAAGGTCTAAATACAAGTA | Cloning; binds 5’end of *mxiE* and adds EcoRI |
| HMIO702 | GGCAAGATCGTCCGTTGTCATAATCGAC | PCR; forward primer for the amplification of the biotin operator from str. M90T |
| HMIO703 | GCCATGGGGCTTCTCCAAAACGTG | PCR; reverse primer for the amplification of the biotin operator from str. M90T |
| HMIO734 | AGAGAGGGTACCTCTTTAAATATCACCGAAAATGAAAGCATCTCTACTG | Cloning; binds 5’end of *ipgC* and adds KpnI |
| HMIO735 | AGAGAGGAGCTCTTACTCCTTGATATCCTGAATTGCGTCCAAG | Cloning; binds 3’end of *ipgC* and adds SacI |

**Table S7.** Plasmids used in this study

| **Plasmid ID** | **Plasmid name** | **Promoter** | **Reference** | **Addgene ID** |
| --- | --- | --- | --- | --- |
| pSU2.1 | pSU2.1 | Constitutive *lac* promoter | (4) | N/A |
| pNHA1 | pSU2.1 *bioID-myc* | Constitutive *lac* promoter | This study | N/A |
| pNHA2 | pSU2.1 *bioID2-myc* | Constitutive *lac* promoter | This study | N/A |
| pNHA3 | pSU2.1 t*urboID-myc* | Constitutive *lac* promoter | This study | N/A |
| pNHA4 | pSU2.1 *miniTurbo-myc* | Constitutive *lac* promoter | This study | N/A |
| pNHA5 | pQE-80L *turboID* | Inducible T5/*lac* promoter | This study | 223647 |
| pNHA6 | pQE-80L *bioID* | Inducible T5/*lac* promoter | This study | 223648 |
| pNHA7 | pQE-80L *birA* | Inducible T5/*lac* promoter | This study | 223649 |
| pNHA8 | pQE-80L *MBP-bccP* | Inducible T5/*lac* promoter | This study | 223650 |
| pNHA9 | pUC18.1rp | Constitutive *rpsM* promoter | This study | 223642 |
| pNHA10 | pUC18.1rp t*urboID-myc* | Constitutive *rpsM* promoter | This study | 223554 |
| pNHA11 | pUC18.1rp *ipgC-turboID-myc* | Constitutive *rpsM* promoter | This study | 223643 |
| pNHA12 | pUC18.1rp t*urboID-myc-ipgC* | Constitutive *rpsM* promoter | This study | 223644 |
| pNHA13 | pUC18.1rp *mxiE-turboID-myc* | Constitutive *rpsM* promoter | This study | 223645 |
| pNHA14 | pUC18.1rp *turboID-myc-mxiE* | Constitutive *rpsM* promoter | This study | 223646 |

**Supplemental Materials Reference**

1. Allaoui A, Mounier J, Prevost MC, Sansonetti PJ, Parsot C. 1992. icsB: a Shigella flexneri virulence gene necessary for the lysis of protrusions during intercellular spread. Molecular Microbiology 6:1605–1616.

2. Menard R, Sansonetti PJ, Parsot C. 1993. Nonpolar mutagenesis of the ipa genes defines IpaB, IpaC, and IpaD as effectors of Shigella flexneri entry into epithelial cells. Journal of Bacteriology 175:5899–5906.

3. Mavris M, Page AL, Tournebize R, Demers B, Sansonetti P, Parsot C. 2002. Regulation of transcription by the activity of the Shigella flexneri type III secretion apparatus. Molecular Microbiology 43:1543–1553.

4. Silué N, Campbell-Valois F-X. 2022. *icaR* and *icaT* are Ancient Chromosome Genes Encoding Substrates of the Type III Secretion Apparatus in Shigella flexneri. mSphere e00115-22.
